# Scalable Graphene Defect Prediction Using Transferable Learning

**DOI:** 10.3390/nano11092341

**Published:** 2021-09-09

**Authors:** Bowen Zheng, Zeyu Zheng, Grace X. Gu

**Affiliations:** 1 Department of Mechanical Engineering, University of California, Berkeley, CA 94720, USA; bowen_zheng@berkeley.edu; 2 Department of Industrial Engineering and Operations Research, University of California, Berkeley, CA 94720, USA; zyzheng@berkeley.edu

**Keywords:** graphene, defects, machine learning, transferable learning, molecular dynamics simulation

## Abstract

Notably known for its extraordinary thermal and mechanical properties, graphene is a favorable building block in various cutting-edge technologies such as flexible electronics and supercapacitors. However, the almost inevitable existence of defects severely compromises the properties of graphene, and defect prediction is a difficult, yet important, task. Emerging machine learning approaches offer opportunities to predict target properties such as defect distribution by exploiting readily available data, without incurring much experimental cost. Most previous machine learning techniques require the size of training data and predicted material systems of interest to be identical. This limits their broader application, because in practice a newly encountered material system may have a different size compared with the previously observed ones. In this paper, we develop a transferable learning approach for graphene defect prediction, which can be used on graphene with various sizes or shapes not seen in the training data. The proposed approach employs logistic regression and utilizes data on local vibrational energy distributions of small graphene from molecular dynamics simulations, in the hopes that vibrational energy distributions can reflect local structural anomalies. The results show that our machine learning model, trained only with data on smaller graphene, can achieve up to 80% prediction accuracy of defects in larger graphene under different practical metrics. The present research sheds light on scalable graphene defect prediction and opens doors for data-driven defect detection for a broad range of two-dimensional materials.

## 1. Introduction

The emergence of nanomaterials research has shown promise in influencing technological applications such as stretchable electronics and supercapacitors [1,2,3,4,5,6]. The existence of defects, however, remains an issue that severely compromises the extraordinary thermal and mechanical properties of various nanomaterials such as graphene [7,8,9,10]. For instance, the Young’s modulus of defect-containing graphene ranges from 0.15 to 0.95 TPa, compared to pristine graphene’s 1 TPa [11,12]. For thermal properties, defects can lower graphene’s thermal conductivity by one to three orders of magnitude [12]. Advanced computational techniques such as molecular dynamics (MD) simulation and density functional theory (DFT) have shed light on the properties of defect-containing graphene [13,14,15,16,17,18]. These techniques require graphene atomic structures as the input, of which the determination is not an easy task and demands taxing experimental procedures.

The development of machine learning approaches enables predictions of target properties using learned patterns from data, and has been gaining momentum in materials prediction, design, and discovery [19,20,21,22,23,24,25,26]. Recently, machine learning has been applied to predict defect distribution [27], mechanical responses [28], chemical compositions [29], viscosity [30], among other nanomaterial properties of importance. However, most machine learning techniques require the size of material systems used in training to be identical to the ones they predict. This limits their application in reality because an identical size of newly encountered materials is by no means guaranteed. It is thereby hoped that the effectiveness of machine learning models be transferable across size and shape to more broadly apply the techniques. In the context of graphene defect prediction, transferable machine learning will enable the prediction of differently sized or shaped graphene sheets trained by data of graphene sheets of a uniform size. In addition, due to the large size, directly simulating graphene used in actual applications can be rather computationally expensive, especially when a large amount of training data is demanded. Transferable machine learning will allow us to use smaller graphene as training data that are more computationally tractable. Hence, a transferable machine learning approach for defect prediction is very much desired.

Another challenge lies in the feature selection. Because whether a certain location on graphene is next to a defect or not is local information (as opposed to global information), selected features need to sufficiently reflect graphene local anomalies. Graphene stiffness, failure strength, and strain, among other collective properties, may not be suitable, because different defect distributions may achieve very similar observations [17]. Thermal vibration, on the other hand, entails local structural information, because the absence of atoms can alter local boundary conditions of mini-oscillators. By probing local thermal vibration behavior, we envision extracting data that reflect the existence of defects in the vicinity of query points.

In this paper, we propose a transferable learning strategy to detect unknown defects in larger graphene sheets using information obtained from a smaller graphene system. Trained by tens of thousands of local vibrational energy distributions of smaller graphene sheets calculated by MD simulations, our machine learning model is used to predict whether certain locations on larger graphene sheets are in the vicinity of any defect. From predicting graphene sheets that contain only one defect to predicting an unknown number of defects with an arbitrary distribution, a logistic regression model is applied, of which the performance is quantified by three practical metrics: total accuracy, true positive rate, and true negative rate. Finally, by adjusting the weights associated with defects and non-defects in the cost function, we aim to find a way of improving the prediction accuracy of defects while maintaining a relatively low false positive rate.

## 2. Materials and Methods

### 2.1. Molecular Dynamics Simulation of Defect-Containing Graphene

**Figure 1:**
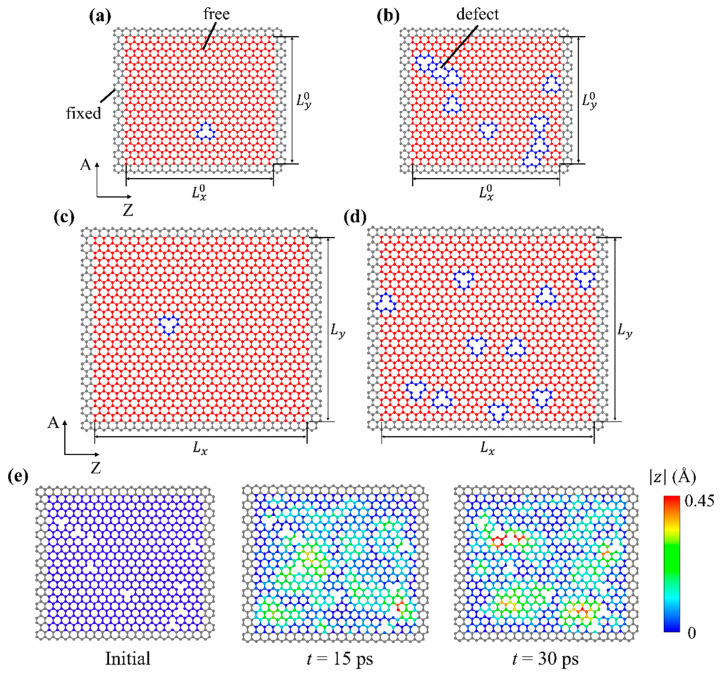
Schematics of defect-containing graphene sheets. Smaller graphene sheets with (**a**) one defect and (**b**) multiple defects for machine learning training. Larger graphene sheets with (**c**) one defect and (**d**) multiple defects for machine learning testing. “A” and “Z” denote the armchair and zigzag directions, respectively. (**e**) An example of vibration amplitude distributions in a defect-containing graphene sheet. Distributions at three different time instances are plotted: before vibration, 15 ps, and 30 ps after the initialization of the NVT ensemble. t denotes the time passed in the NVT ensemble where the vibrational responses are recorded.

Geometries of defect-containing monolayer graphene sheets used in this study are shown in Figure 1 (defects highlighted in blue). Two different sizes of graphene sheets were constructed: smaller ones for training and larger ones for testing. Figure 1a,b illustrate small graphene sheets, sized $L_{x}^{0}$ by $L_{y}^{0}$, where $L_{x}^{0}=43.6\;Å$, $L_{y}^{0}=39.2\;Å$; Figure 1c,d illustrate larger graphene sheets, sized $L_{x}$ by $L_{y}$, where $L_{x}=58.2\;Å$, $L_{y}=51.8\;Å$. “A” and “Z” denote the armchair and zigzag directions of graphene, respectively. On each edge of the graphene sheet, a width of three atoms was fixed (colored gray), while the rest of the graphene sheet could vibrate without enforced restriction (colored red). If defect-free, the smaller graphene sheet contained 722 vibrating atoms, while the larger graphene sheet contained 1250 vibrating atoms. The out-of-plane displacement z of atoms was used to quantify vibration. In Figure 1a,c, the graphene sheets contained only one defect, of which the location was uniformly random throughout the vibrating graphene domain; in Figure 1b,d, the graphene sheets contained multiple defects where the number of defects followed a uniform distribution from 1 to 10, and the locations were also uniformly random throughout the vibrating graphene domain.

MD simulations were used to compute the vibration of graphene, performed using the open-source code LAMMPS (Large-scale Atomic/Molecular Massively Parallel Simulation) [31]. The full atomic description was used. Interactions between atoms were modeled by an Adaptive Intermolecular Reactive Empirical Bond-Order (AIREBO) potential [32], including short-ranged, long-ranged, and torsional interactions, as expressed below:

(1) $$E=E^{\mathrm{REBO}}+E^{\mathrm{LJ}}+E^{\mathrm{tors}}$$

where E is the total system energy; $E^{\mathrm{REBO}}$, $E^{\mathrm{LJ}}$, and $E^{\mathrm{tors}}$ are energy components corresponding to the REBO (short-ranged), Lennard-Jones (long-ranged), and torsional potentials.

AIREBO potential has been widely used to study the mechanical behavior of defect-containing graphene [18,33,34,35]. A timestep of 1 femtosecond was used. Periodic boundary condition was applied to the two in-plane dimensions, while the height of the simulation box was fixed. For fixed atoms near the graphene edges, the atom displacement in all three directions was set to zero. Each atom in the vibrating region was initiated with a random velocity, and for each individual graphene sheet the seed of the random number generator was different. This practice ensured that even if two graphene sheets contained the same number of defects at the same locations (meaning the two graphene sheets were governed by the same physics) their mechanical responses were not numerically identical, thus preventing data duplication. Graphene sheets were firstly relaxed in the isothermal–isobaric (NPT) ensemble at temperature T=300 K and pressure p=0 for 25 picoseconds to eliminate stress. Then, the graphene sheet was stretched biaxially to 1% strain in the canonical (NVT) ensemble at T=300 K, with a strain rate of 10^9^ s^−1^. The small tensile pre-strain was applied to imitate the experimental setup of graphene vibration in [36]. Finally, the pre-strained graphene sheet was set to vibrate in the NVT ensemble at T=300 K for 30 picoseconds, during which the out-of-plane displacement of all vibrating atoms were extracted for subsequent data processing. The sampling frequency of atom trajectories was 20 THz. Using the graphene sheet in Figure 1b as an example, the distributions of vibration amplitudes 15 ps and 30 ps after the initialization of the NVT ensemble are plotted in Figure 1e. The initial graphene configuration is also provided for comparison.

### 2.2. Machine Learning

**Figure 2:**
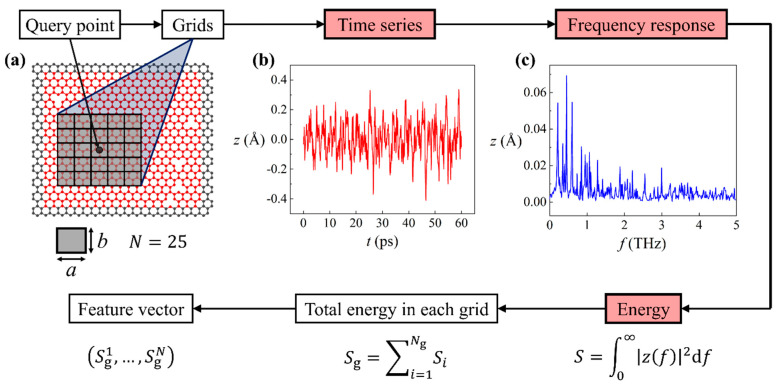
Data preparation procedure for machine learning. Boxes in red are properties of each individual atoms. (**a**) Illustration of query point and grid construction. (**b**) Displacement time series z(t) and (**c**) the corresponding frequency response z(f) of a vibrating atom in the graphene sheet.

The data preparation procedure for machine learning is shown in the flowchart in Figure 2. First, one or multiple query points were assigned on the graphene sheet. Grids were subsequently constructed surrounding the query point, as illustrated in Figure 2a. The query point resides at the center of the center grid. Each grid is sized a-by-b, where $a:b=L_{x}^{0}:L_{y}^{0}$, making the length-to-height ratio of grids equal to the length-to-height ratio of the smaller graphene sheet. Because of the fixed ratio, a single variable a is sufficient to depict the grid size. In the present study, we considered 9-grid (3 by 3) and 25-grid (5 by 5) approaches. The accuracy comparison of the two approaches will be conducted. Next, we computed the vibration of atoms in these grids, detailed as follows: The time series of out-of-plane displacement z(t) of each atom is computed by MD simulations, of which an example is provided in Figure 2b. Then, a fast Fourier transformation is performed on z(t) to deduce its frequency response z(f), as shown in Figure 2c.

Our next goal was to associate the vibration of each atom to a scalar. To this end, based on the frequency response we calculated the energy as $S=\int_{0}^{\infty }{\left|z\left(f\right)\right|}^{2}df$. Afterwards, the total energy in each grid $S_{g}$ was computed as $S_{g}=\sum_{i=1}^{N_{g}}S_{i}$, where i is the index of atom in the grid, and $N_{g}$ is the total number of atoms in the grid. Finally, a feature vector is formed as $\left(S_{g}^{1},\ldots ,S_{g}^{N}\right)$, where N is the number of grids (N=9 or 25). Up to this point, we associated each query point with a feature vector that quantifies vibration properties in the grids. The label Y was determined by the existence of defects in the center grid: if there was one or more defects in the center grid, label Y=1; otherwise Y=0. It is a natural choice to apply a logistic regression model to predict binary classes using feature vectors. The loss function of logistic regression can be expressed as:

(2) $$L\left(\hat{Y},Y\right)=-Y\ln\hat{Y}-\left(1-Y\right)\ln\left(1-\hat{Y}\right)$$

where $\hat{Y}$ is the predicted label. 

The cost function can be expressed as:

(3) $$J=\sum_{i=1}^{n}L\left({\hat{Y}}_{i},Y_{i}\right)$$

where i is the index of one data point, and n is the total number of data points. 

In this study, logistic regression was performed using the machine learning library Scikit-Learn [37].

## 3. Results and Discussion

For our binary classification problem, we quantified prediction accuracy by the following three metrics: total accuracy (TA), true positive rate (TPR), and true negative rate (TNR), expressed below:

(4) $$\mathrm{TA}=P\left(\hat{Y}=1|Y=1\right)P\left(Y=1\right)+P\left(\hat{Y}=0|Y=0\right)P\left(Y=0\right)$$

(5) $$\mathrm{TPR}=P\left(\hat{Y}=1|Y=1\right)$$

(6) $$\mathrm{TNR}=P\left(\hat{Y}=0|Y=0\right)$$

where $\hat{Y}$ is the predicted label; P( ) is the probability notation, and P(·|·) denotes the conditional probability.

### 3.1. One-Defect Scenarios

**Figure 3:**
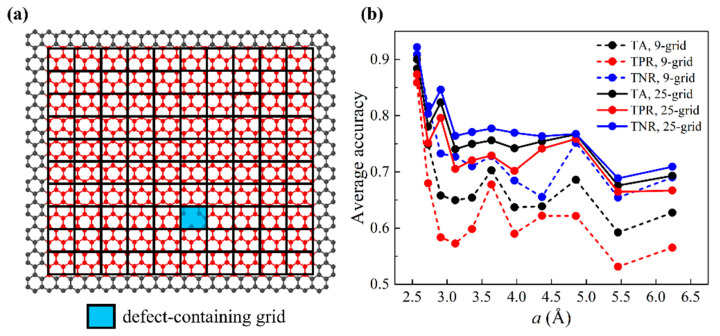
Machine learning results of validation sets for one-defect scenarios. (**a**) Schematic of discretized graphene domain for one-defect graphene sheets. The entire vibrating graphene domain is discretized by an M-by-M mesh (in this case, M=10, a=4.37 Å). Defect-containing grid is highlighted in light blue. (**b**) Validation accuracies (TA, TPR, TNR) as a function of the grid size a for 9-grid and 25-grid approaches.

The prediction of one-defect graphene sheets was studied first. The preparation of training data based on the smaller graphene is detailed as follows: First, we discretized the entire vibrating graphene domain with an M-by-M mesh, as shown in Figure 3a. M is an integer parameter ranging from 7 to 17, giving values of a from 6.23 Å to 2.57 Å. This practice aims to thoroughly scan the graphene domain during training. The ratio $a:b=L_{x}^{0}:L_{y}^{0}$ ensures that the numbers of grids in the two orthogonal directions (i.e., the armchair and the zigzag directions) are equal. To prepare unbiased training data for our binary classification model, we equalized the numbers of Y=1 data and Y=0 data. Here, we purposefully selected the defect-containing grid as the center grid and constructed the rest of the grids around it. Then, we randomly selected another grid as a center grid, which was bound to be defect-free. Hence, each graphene provides two sets of training data, and the numbers of Y=1 data and Y=0 data are equal. The total number of one-defect graphene sheets for training is 10,148. However, not all these graphene sheets were used to construct training data—graphene sheets that contained a near-edge/-corner defect were excluded due to different lengths of feature vectors. For the 9- (25-) grid approach, graphene sheets whose defect-containing center grids that were within one (two) grid(s) to graphene edges were excluded. We addressed a total of five types of near-edge/-corner defects separately and assigned the results to the Supplementary Material. Data were randomly split into 9:1 for training and validation. Validation accuracies were based on the average of three different splits. Figure 3b shows validation accuracies (TA, TPR, TNR) as a function of grid size a. The results of 9-grid and 25-grid approaches are also compared. It is shown that as a increases, all three accuracy metrics decrease. This suggests that smaller grids give rise to higher training accuracies. In addition, 9-grid and 25-grid approaches have similar trends and the 25-grid approach strikes higher accuracy in all three metrics. Hereafter, machine learning results will be based on only the 25-grid approach for simplicity. We also tested out specifying the query point location $x/L_{x}^{0}$ and $y/L_{y}^{0}$ in the feature vectors, where (x,y) are the coordinates of the query point. It is shown that adding these two features has little impact on the accuracies of either 9-grid and 25-grid approaches, as illustrated in Figure S1 in the Supplementary Material.

**Figure 4:**
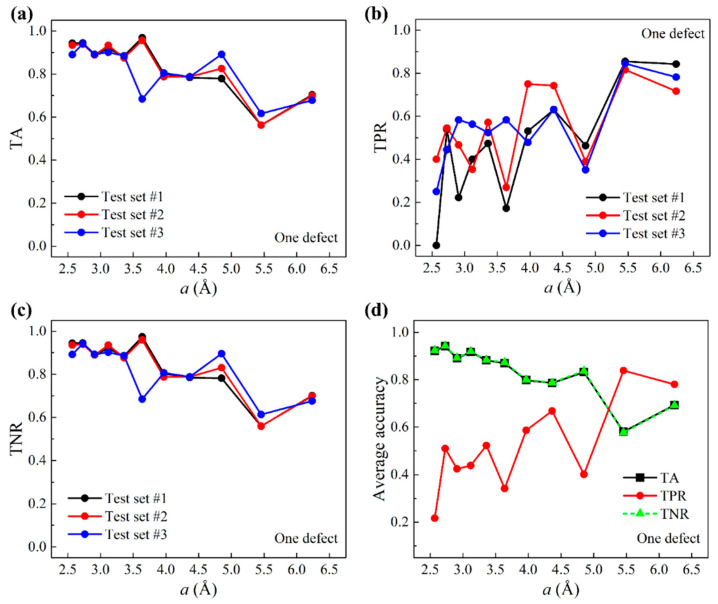
Test accuracies for one-defect scenarios. (**a**) TA, (**b**) TPR, and (**c**) TNR as a function of the grid size a based on three test sets. (**d**) Average accuracies over all three test sets as a function of a.

The preparation of test data based on larger graphene sheets differed from the training data, as is outlined below. The number of larger graphene sheets for machine learning testing is 875. Each provided five randomly chosen query points that were at least two grids away from graphene edges. The prediction of query points within two grids to graphene edges is assigned to the Supplementary Material. Because of the random selection, center grids that contained defects were outnumbered by defect-free center grids, different from the previous training data where the numbers of defective and defect-free center grids were equal. Test accuracies based on three distinct test sets are presented in Figure 4. Figure 4a–c show TA, TPR, and TNR as a function of the grid size a, respectively. It is shown that both TA and TNR decrease as a increases. TPR, on the other hand, increases as a increases, which differs from the trend of TPR in the validation result. This suggests that for TPR, our machine learning model is subject to a high-variance issue; this shows that it is more ideal to choose moderately larger grid sizes for this system. In all, TA, TPR, and TNR, the results of three test sets show good consistency. Figure 4d shows the average accuracies of test results. TA and TNR appear to coincide. This is because in the test data, label Y=0 data take on more than 99% of the total data.

### 3.2. Multiple-Defect Scenarios

With the successful implementation of predicting one-defect graphene sheets, we move on to the more complex multiple-defect cases. The total number of smaller multiple-defect graphene sheets for training is 10,108. The same as one-defect scenarios, the vibrating graphene domain was discretized by an M-by-M mesh. Defect-containing grids that were more than two grids away from any graphene edge were used as center grids. To equalize Y=1 data and Y=0 data, an equal number of defect-free grids were randomly selected as center grids. These data were randomly split into 9:1 for training and validation. Validation accuracies as a function of the grid size a are provided in Figure S2 in the Supplementary Material.

**Figure 5:**
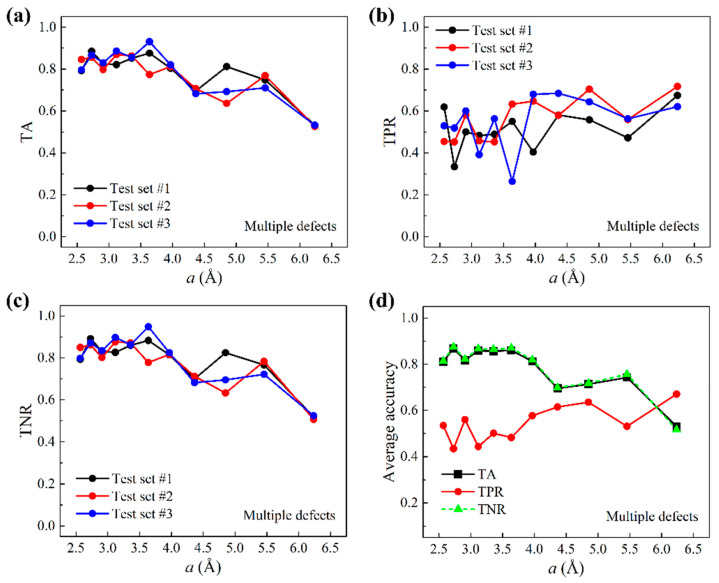
Test accuracies for multiple-defect scenarios. (**a**) TA, (**b**) TPR, and (**c**) TNR as a function of the grid size a based on three test sets. (**d**) Average accuracies over all three test sets as a function of a.

The preparation of multiple-defect test data based on the larger graphene sheets is outlined below. A total of 621 larger graphene sheets were used and each provided five randomly chosen query points at least two grids away from graphene edges. The prediction of query points within two grids to graphene edges is shown in Figures S3–S7 in the Supplementary Material. Because of the random selection, grids that contain defects were outnumbered by defect-free grids. Figure 5a–c show TA, TPR, and TNR as a function of the grid size a. It is shown that both TA and TNR decrease as a increases, and that TPR increases as a increases. Compared to one-defect scenarios, TPR is lower in general but is higher for small grid sizes, indicating less susceptibility to the high-variance-related issue. Figure 5d presents the average accuracies of test results. TA and TNR appear to coincide. This is because in the test data, label Y=0 data take on more than 98% of the total data. The above results of one-defect and multiple-defect scenarios indicate that, trained by local thermal vibration properties of uniformly sized small graphene sheets, our machine learning model is able to predict the existence of defects at all locations on unseen larger graphene sheets.

### 3.3. Weighted Cost Function

It is desired that the prediction accuracy of a defect in larger graphene sheets can be further improved, and that the fine-tuning of TPR and TNR is enabled as we emphasize the prediction of defects or non-defects as needed. A solution to this can be offered by a weighted version of the cost function, expressed as:

(7) $$J=\sum_{i=1}^{n}w_{i}L\left({\hat{Y}}_{i},Y_{i}\right)$$

where:

(8) $$w_{i}=\{\begin{array}{ll} w_{0}, & \;Y_{i}=0\\ w_{1}, & \;Y_{i}=1 \end{array}$$

**Figure 6:**
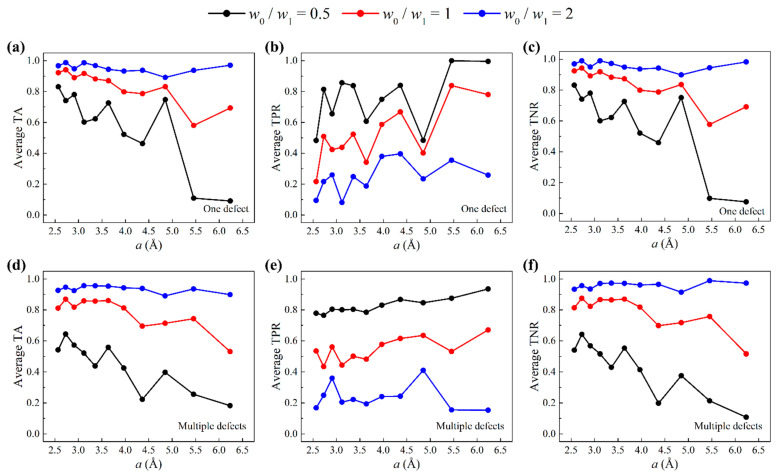
Accuracies when a weighted cost function is used (weight ratios $w_{0}/w_{1}=0.5,\;1,\;2$). Average (**a**) TA, (**b**) TPR, and (**c**) TNR of three test sets as a function of grid size a for one-defect graphene sheets. Average (**d**) TA, (**e**) TPR, and (**f**) TNR of three test sets as a function of grid size a for multiple-defect graphene sheets.

By tuning the weights $w_{0}$ and $w_{1}$, we change the significance attached to the correct prediction of defects and non-defects. Concretely, if $w_{0}/w_{1}<1$, accuracy of defects is emphasized; if $w_{0}/w_{1}>1$, accuracy of non-defects is emphasized; if $w_{0}/w_{1}=1$, defects and non-defects are equally emphasized, which is the default for all previous machine learning results. Here, we compare the prediction accuracies using a weighted cost function with weight ratios $w_{0}/w_{1}=0.5,\;1,\;2$. Figure 6a–c show the average test accuracies of one-defect cases. Figure 6d–f show the average test accuracies of multiple-defect cases. In all cases, TPR increases as $w_{0}/w_{1}$ decreases, while TNR increases as $w_{0}/w_{1}$ increases. For all three metrics, there is a tradeoff between TPR and TNR, giving us the opportunity to emphasize one label over another. Notably, the weight ratio $w_{0}/w_{1}=2$ gives the highest TA. This is because in both one-defect and multiple-defect test sets, Y=0 data outnumber Y=1 data. By applying weighted cost function, we gain the ability to improve the prediction accuracy of defects while maintaining a relatively low false positive rate.

### 3.4. Discussion

In this work, due to the randomized atom removal process of defect creation, most defects assigned to graphene sheets are unreconstructed single vacancies. Even though unreconstructed single vacancies have been observed in experiments [38] and studied in other computational work as the main defect type [35,39], graphene displays an incredibly rich restructuring capability which gives rise to reconstructed vacancy defects, for instance, through a Jahn–Teller distortion [40]. In our future work, we will take into account more types of defects, including reconstructed single vacancies, bivacancies, divacancies, and Stone–Wales vacancies, among others that have a potentially lower formation energy [41]. Our proposed machine learning approach is believed to be applicable to all types of vacancies, because these vacancies give rise to structural anomalies that can cause changes to local vibration properties. Additionally, we do not rule out the existence of larger defects, which arise when removed atoms are next to each other (see for an example Figure S8 in the Supplementary Material). More accurate representations of reconstructed larger defects suggested by DFT and experimental observations will be devoted to our future work. For experimental possibility, Raman spectroscopy, despite the ability to identify the number and nature of defects in graphene, is not particularly suited for pinpointing defect locations. As an option, vibrational energy distribution used as the machine learning input can be readily obtained using a scanning force microscope [42] or interferometry [36].

## 4. Conclusions

In summary, we present a transferable learning strategy to detect unknown defects in larger graphene sheets using information obtained from only a smaller graphene system. Trained by tens of thousands of local vibrational energy distributions in uniformly sized smaller graphene sheets from MD simulations, our logistic regression model can predict defects in unseen larger graphene sheets with satisfactory accuracies for both one-defect and multiple-defect graphene sheets, quantified by three practical metrics: TA, TPR, and TNR. Through adjusting the weights associated with defects and non-defects in the cost function, we achieve an improvement in the prediction accuracy of defects while maintaining a relatively low false positive rate. The present research sheds light on scalable graphene defect prediction and opens doors for accelerated data-driven defect detection of a broad range of two-dimensional materials. Future work includes the further improvement of prediction accuracies utilizing more advanced machine learning models as well as the prediction of other types of defects such as substitutions and dislocations, among others.

## Data Availability

Data are available from the corresponding author upon request.

## Supplementary Materials

The following are available online at https://www.mdpi.com/article/10.3390/nano11092341/s1, Figure S1: Validation accuracies with and without query point locations, Figure S2: Validation accuracies of multiple-defect graphene sheets, Figure S3: Validation and test accuracies of Type 1 near-edge/-corner defects, Figure S4: Validation and test accuracies of Type 2 near-edge/-corner defects, Figure S5: Validation and test accuracies of Type 3 near-edge/-corner defects, Figure S6: Validation and test accuracies of Type 4 near-edge/-corner defects, Figure S7: Validation and test accuracies of Type 5 near-edge/-corner defects, Figure S8: An example of graphene sheet that contains a larger defect.
