# Supplementary material for: Scalable Graphene Defect Prediction Using Transferable Learning
Source: Nanomaterials (Basel). 2021 Sep 9;11(9):2341. doi: 10.3390/nano11092341 (PMC8472110; doi:10.3390/nano11092341)
Supplement: Supplementary file 1 [file nanomaterials-11-02341-s001.zip › nanomaterials-1341002-supplementary.pdf]

nanomaterials

Supplementary Materials

Scalable Graphene Defect Prediction Using Transferable Learning

Bowen Zheng[1], Zeyu Zheng[2] and Grace X. Gu[1,*]

[1] Department of Mechanical Engineering, University of California, Berkeley, Berkeley, CA 94720, USA
[2] Department of Industrial Engineering and Operations Research, University of California, Berkeley, Berkeley, CA 94720, USA
* Correspondence: ggu@berkeley.edu

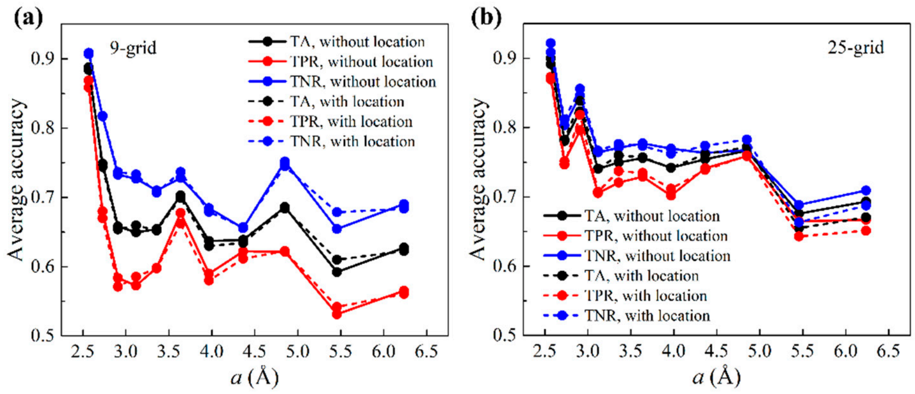

Figure S1. Validation accuracies with and without query point locations. (a) 9-grid and (b) 25-grid approaches.

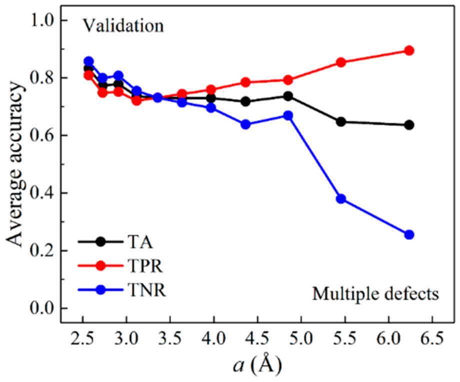

Figure S2. Validation accuracies of multiple-defect graphene sheets.

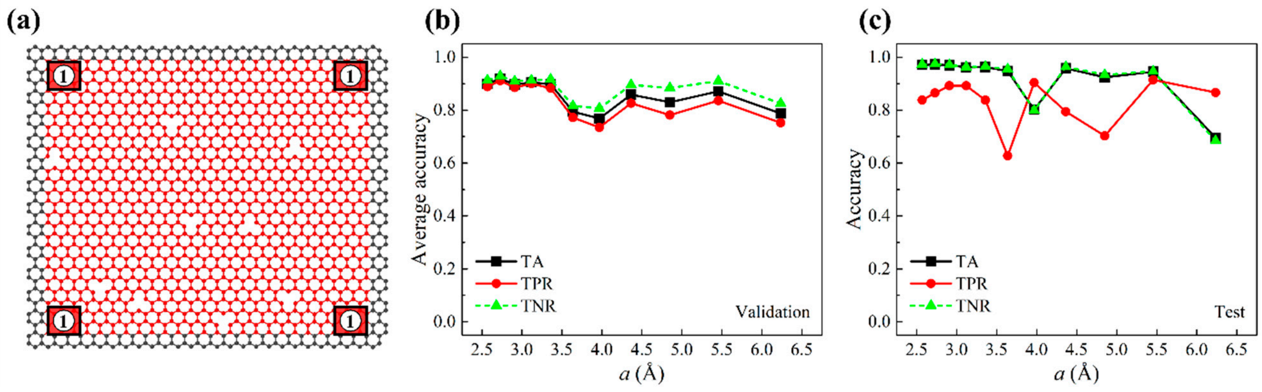

Figure S3. Validation and test accuracies of Type 1 near-edge/-corner defects. The length of feature vectors is 9. (a) Illustration of Type 1 near-edge/-corner defects. (b) Validation and (c) test accuracies.

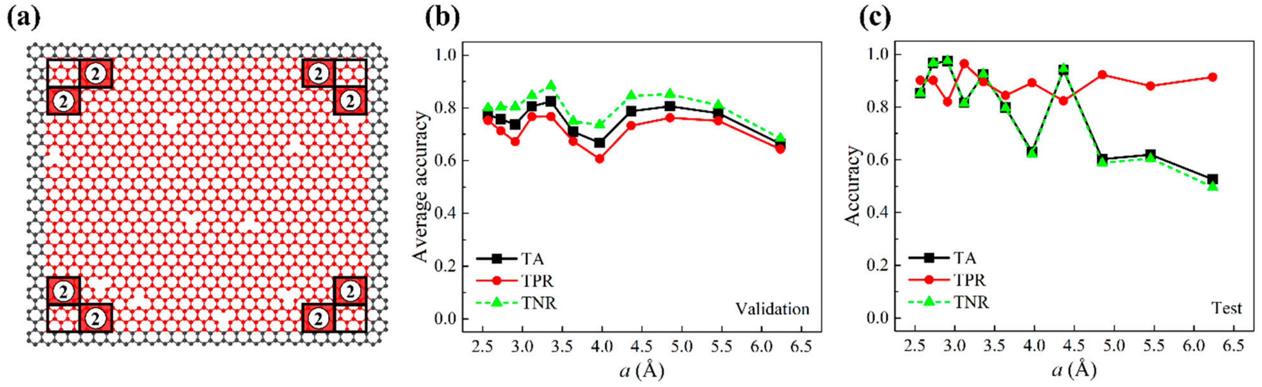

Figure S4. Validation and test accuracies of Type 2 near-edge/-corner defects. The length of feature vectors is 12. (a) Illustration of Type 2 near-edge/-corner defects. (b) Validation and (c) test accuracies.

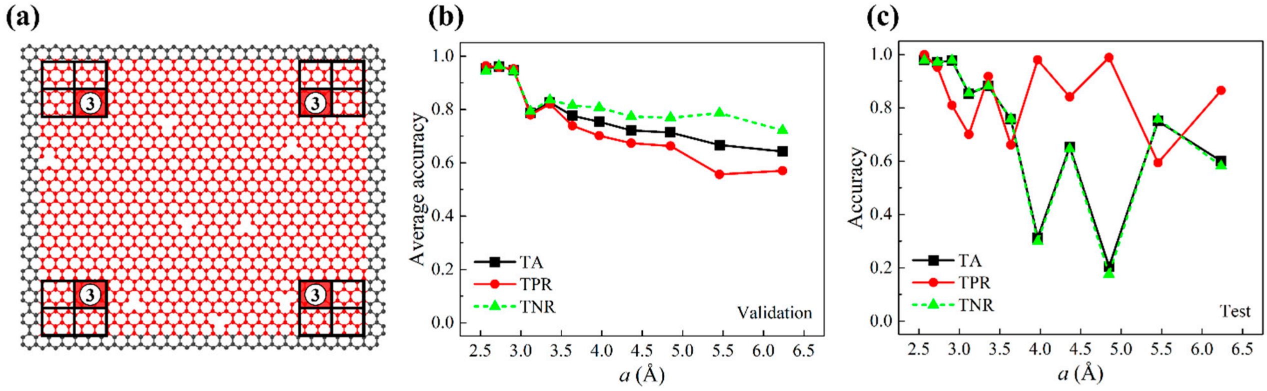

Figure S5. Validation and test accuracies of Type 3 near-edge/-corner defects. The length of feature vectors is 16. (a) Illustration of Type 3 near-edge/-corner defects. (b) Validation and (c) test accuracies.

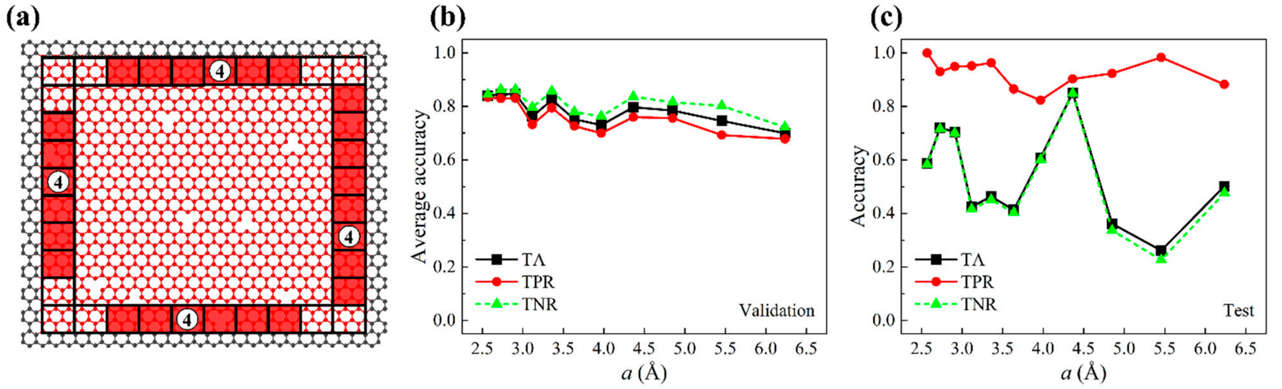

Figure S6. Validation and test accuracies of Type 4 near-edge/-corner defects. The length of feature vectors is 15. (a) Illustration of Type 4 near-edge/-corner defects. (b) Validation and (c) test accuracies.

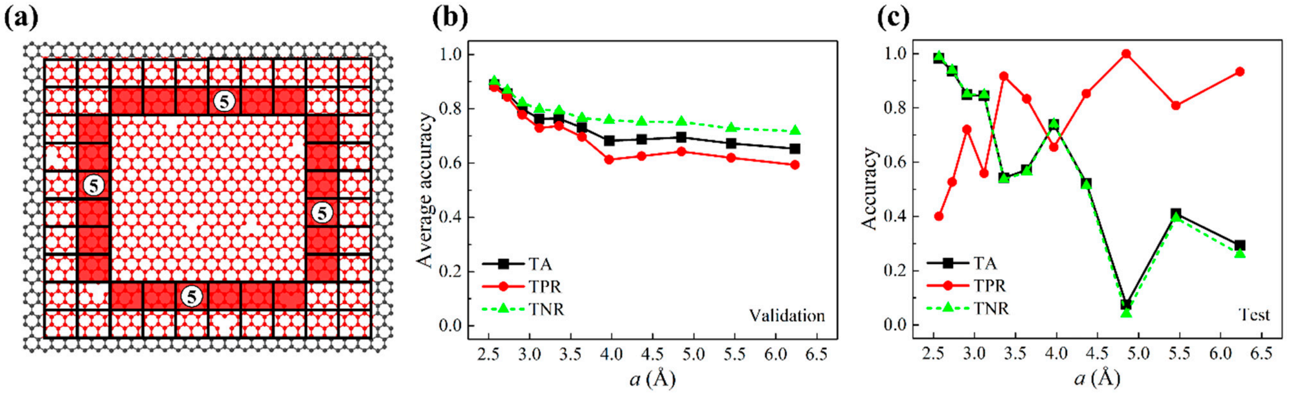

Figure S7. Validation and test accuracies of Type 5 near-edge/-corner defects. The length of feature vectors is 20. (a) Illustration of Type 5 near-edge/-corner defects. (b) Validation and (c) test accuracies.

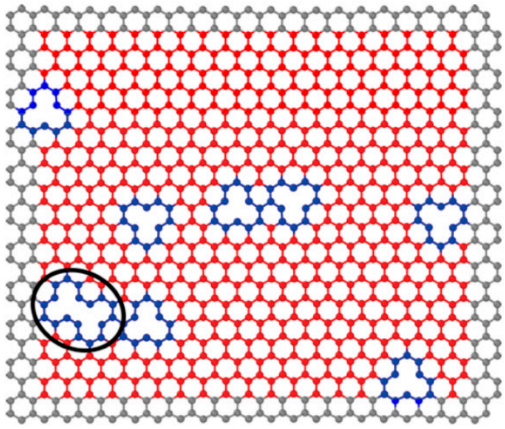

Figure S8. An example of graphene sheet that contains a larger defect (circled).
